# Supplementary material for: Detection of viral RNAs at ambient temperature via reporter proteins produced through the target-splinted ligation of DNA probes
Source: Nat Biomed Eng. 2023 May 4;7(12):1571–82. doi: 10.1038/s41551-023-01028-y (PMC10727988; doi:10.1038/s41551-023-01028-y)
Supplement: Supplementary file 1 — Supplementary figures and tables, and unprocessed lateral-flow strips. [file 41551_2023_1028_MOESM1_ESM.pdf]

nature biomedical engineering

Article https://doi.org/10.1038/s41551-023-01028-y

Detection of viral RNAs at ambient temperature via reporter proteins produced through the target-splinted ligation of DNA probes

In the format provided by the authors and unedited

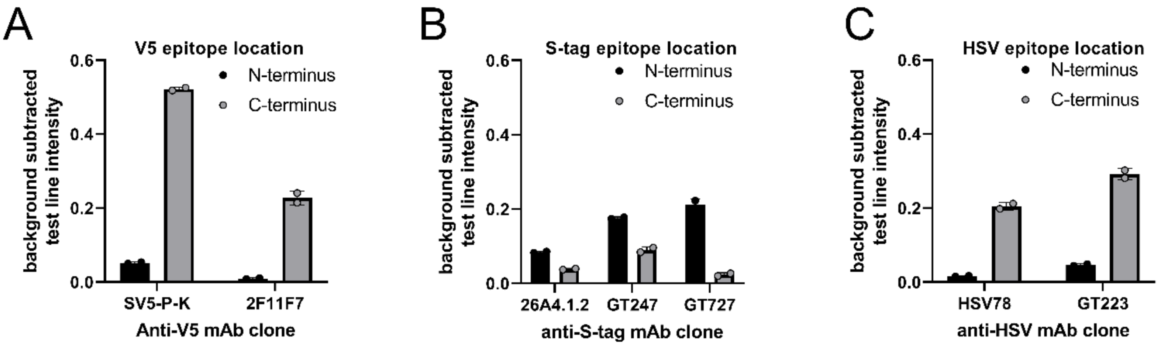

Supplementary Fig. 1 l Epitope location on dual epitope peptide. a, Expression cassettes encoding for the V5 epitope on the C-terminus of the expressed dual epitope peptide yielded stronger test lines than when expressed on the N-terminus. **b,** Expression cassettes encoding for the S-tag epitope on the N-terminus of the expressed dual epitope peptide yielded stronger test lines than when expressed on the C-terminus. **c,** Expression cassettes encoding for the HSV epitope on the C-terminus of the expressed dual epitope peptide yielded stronger test lines than when expressed on the N-terminus. Plotted data represent background subtracted test line intensity of two technical replicates of independent expression reactions and their mean.

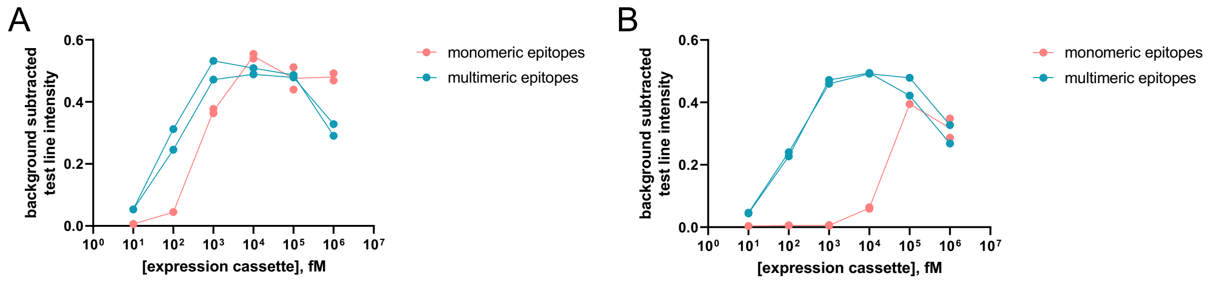

Supplementary Fig. 2 l Multimeric epitopes improve LF detection of dual epitope peptide. a, 10-fold fewer expression cassettes yield detectable dual epitope peptides in PURExpress when encoding tandem epitopes (3XFLAG, Twin-Strep-tag®) than when encoding single epitopes (1xFLAG, Strep-tagII). **b,** Similarly, 1000-fold fewer expression cassettes yield detectable dual epitope peptides in NEBExpress when encoding tandem epitopes (3XFLAG, Twin-Strep-tag®) than when encoding single epitopes (1xFLAG, Strep-tag®II). Plotted data represent background subtracted test line intensity of two technical replicatesof independent expression reactions.

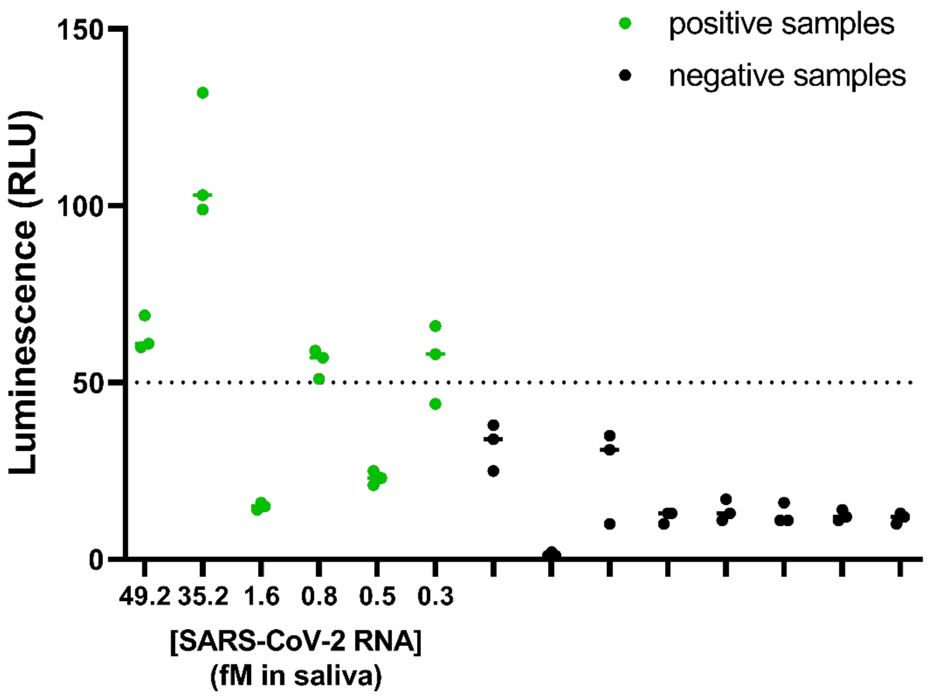

Supplementary Fig. 3 | Luminescent INSPECTR probe for detection of RNA target in clinical sample matrix. Plotted data points represent the one-hour luminescence values detected from the quantitative workflow without amplification and nanoluciferase probe sets, tested on six clinical SARS-CoV-2 positive samples (target concentration measured by qPCR) and eight negatives. The entire workflow was performed in triplicate for all clinical samples. Setting the luminescence cutoff at 50 RLU, the assay successfully detects all samples quantified greater than 10 fM with no false positives. Our workflow included a brief 30-second lysis step to isolate RNA from saliva (see Methods). Plotted data represent background subtracted test line intensity of three technical replicates of independent ligation-expression reactions and their mean.

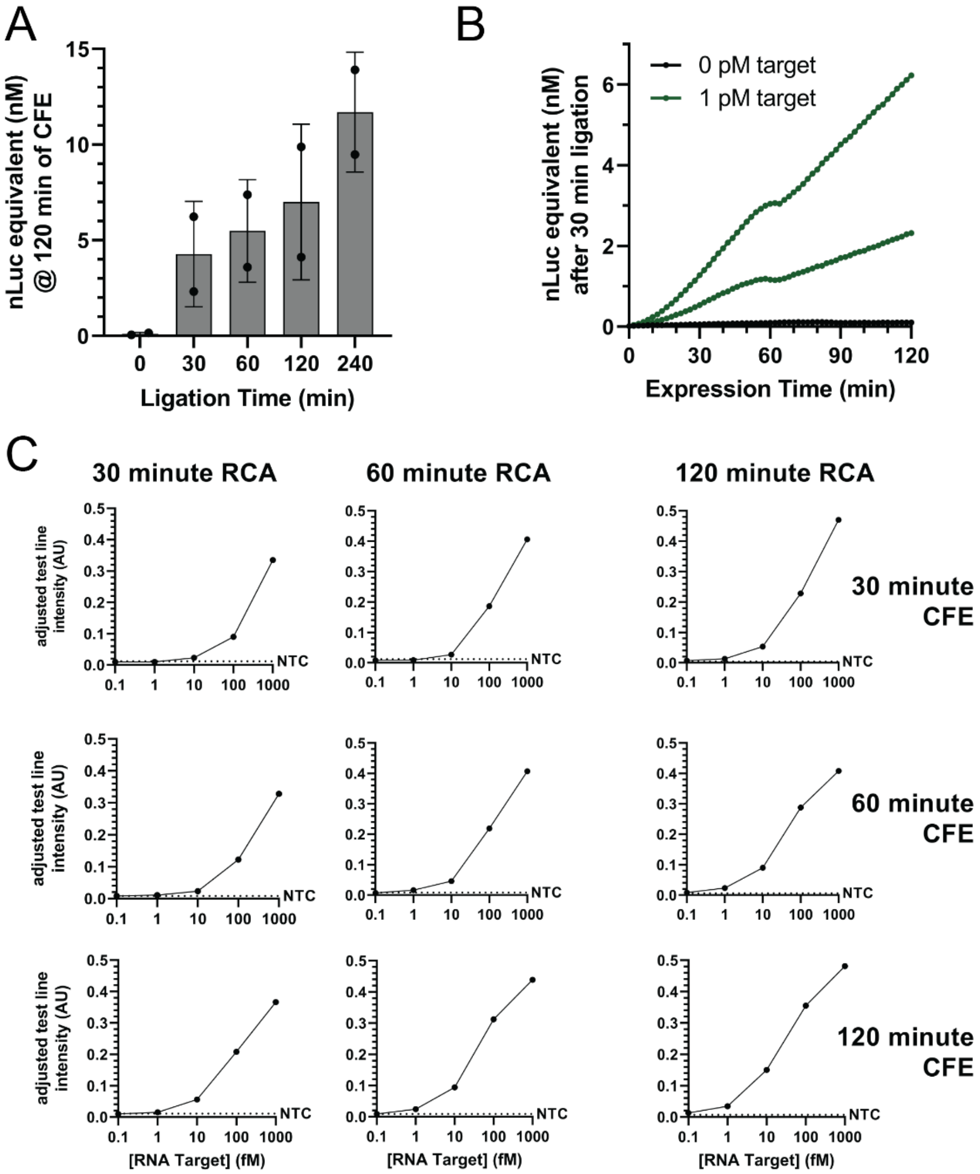

Supplementary Fig. 4 | Reducing the time to result by shortening assay steps also reduces INSPECTR's sensitivity. a, Ligation time-course at a fixed cell-free expression time shows the final signal is roughly linear with target concentration. Assay performed using HiBiT probes and 1 pM of mock SARS-CoV-2 RNA target. Plotted data represent luminescence from of two technical replicates of independent ligation-expression reactions and their mean. **b**, Expression time-course at fixed ligation time of 30 minutes from the same assay. **c**, Covariation of RCA and expression time for dual-epitope peptide expression shows that longer times for both improves the detection limit. Pre-circularized probes (prepared using CircLigase treatment of DNA ultramers) at the indicated concentrations were used as the template for amplification. Plotted data represent single replicates of the individual time pairings.

Supplementary Table 1 I Capabilities enabled by each explored reporter protein

Expressed reporter	Visual Readout	Sensitive	Multiplexed	Quantitative	Time-to-result	Temperature
LacZ alpha	Yes	No	No	No	4 hours	Ambient
nLuc	No	Yes	No	Yes	1 hour	Ambient
Dual-epitope peptide	Yes	No	Yes	No	3 hours	Ambient
Dual epitope peptide (RCA- enhanced)	Yes	Yes	No	No	5 hours	Ambient

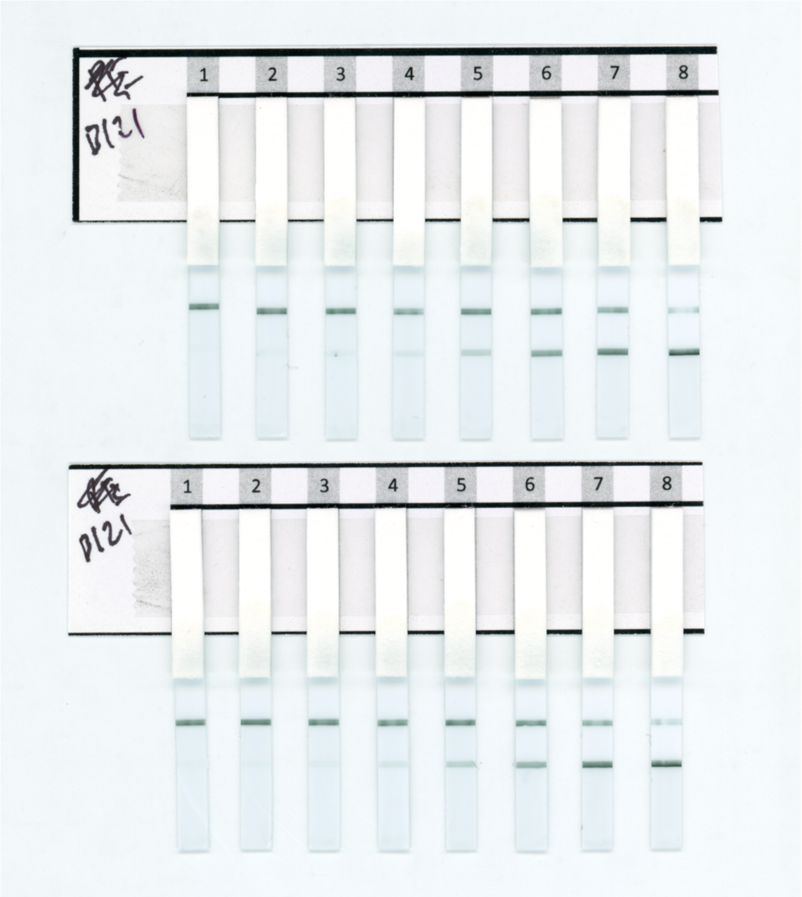

Unprocessed lateral flow strips for Fig. 2b, in-house expression system product.

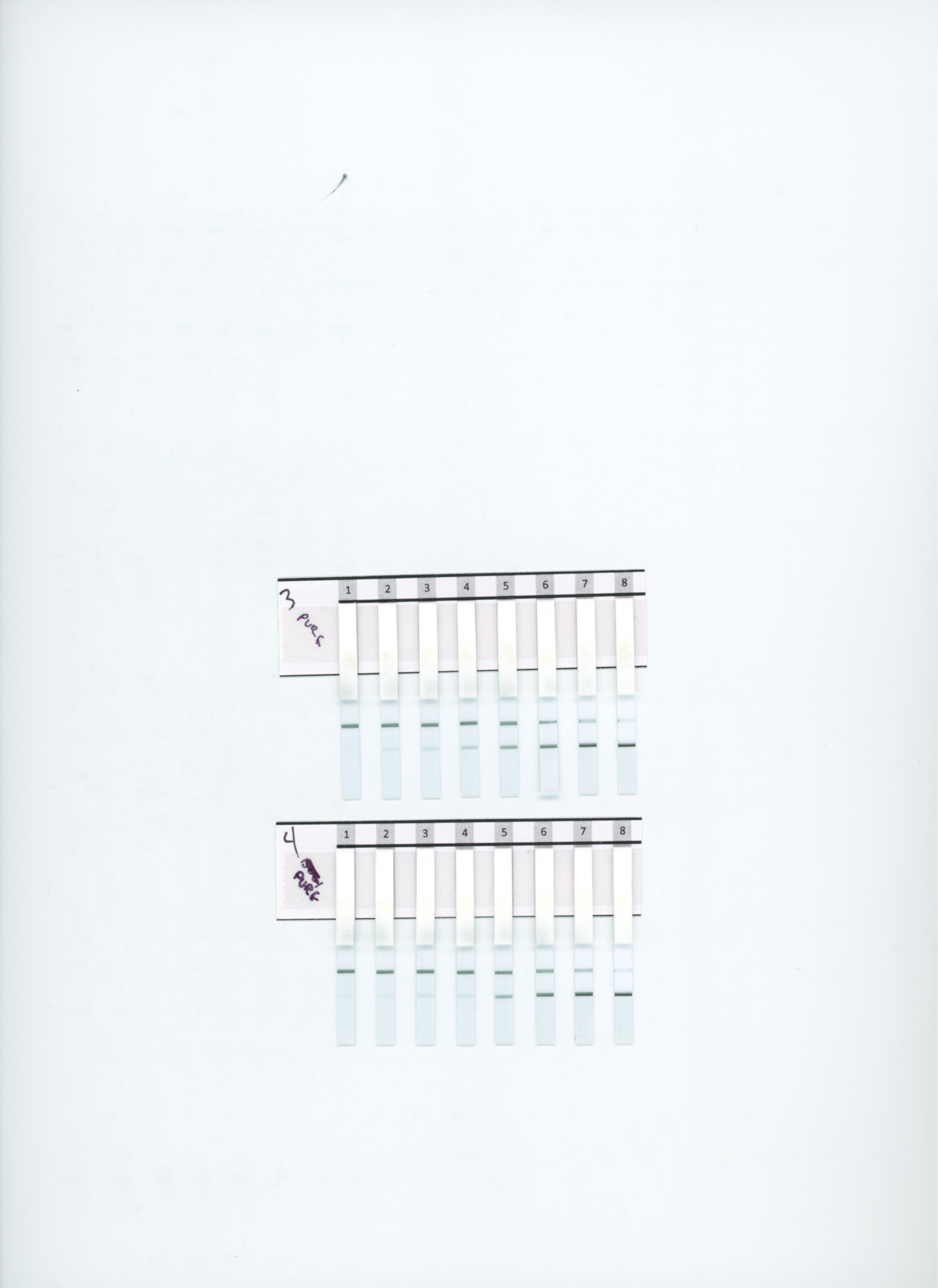

Unprocessed lateral flow strips for Fig. 2b, PURExpress In Vitro Protein Synthesis Kit product.

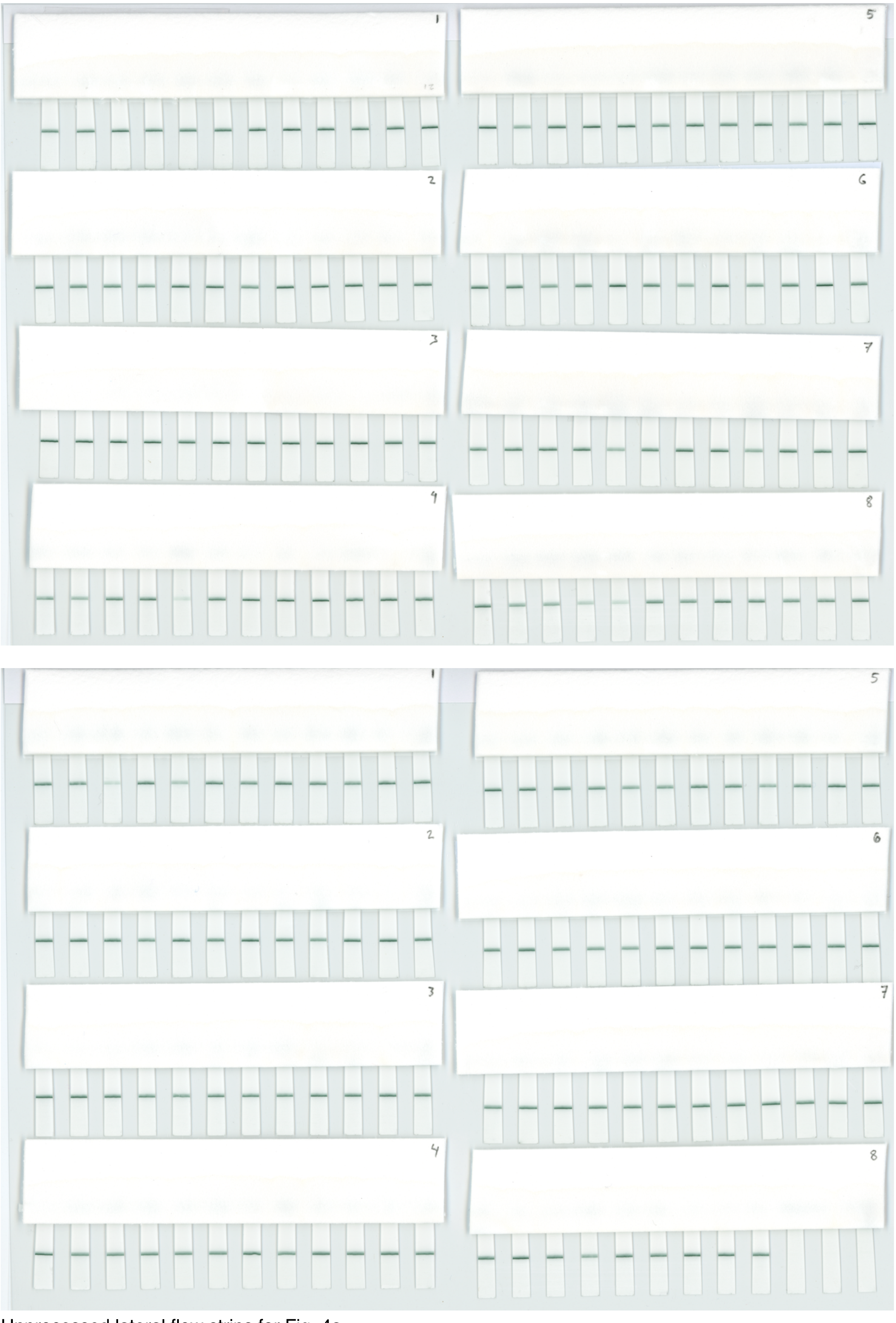

Unprocessed lateral flow strips for Fig. 4a.

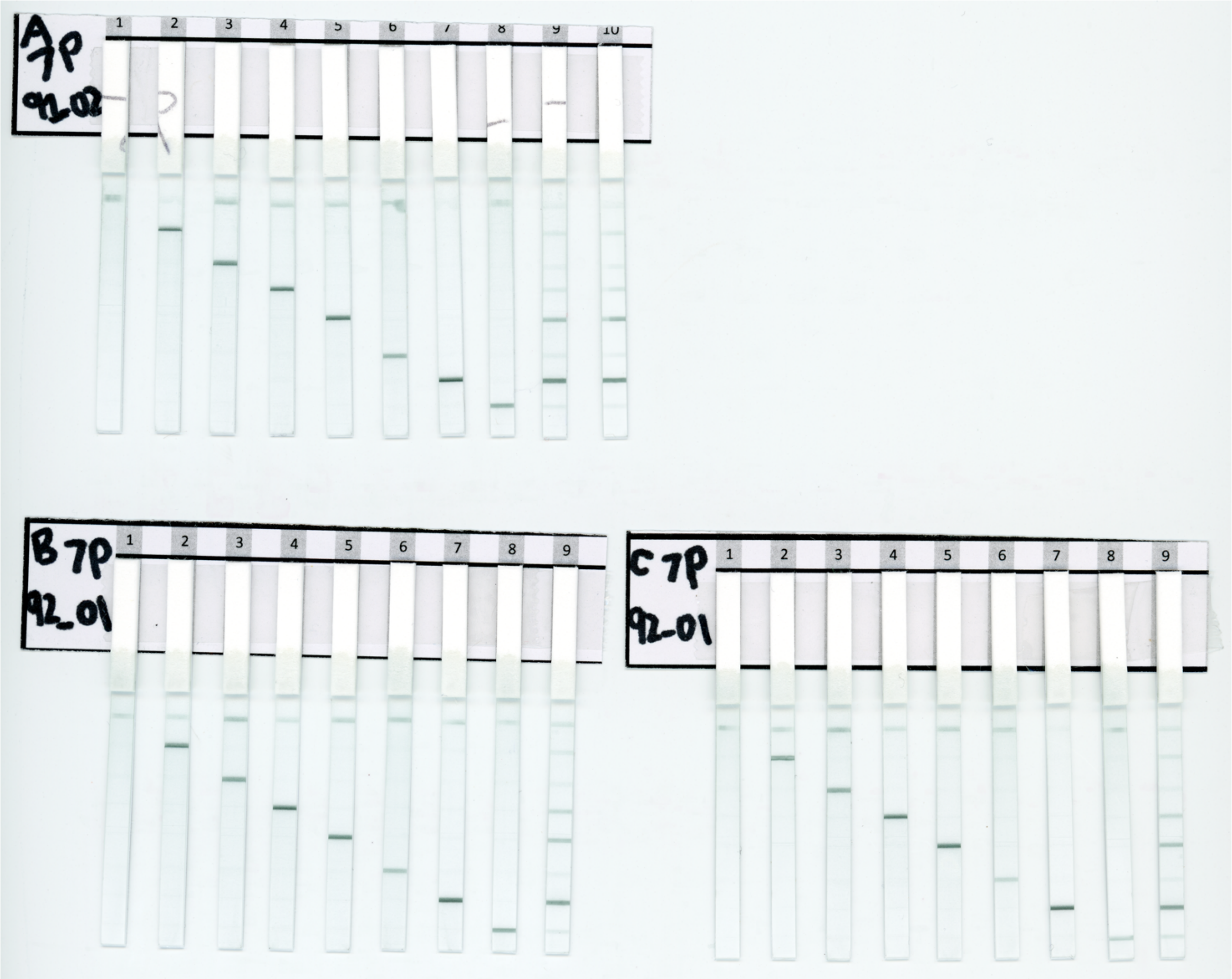

Unprocessed lateral flow strips for Fig. 4b.

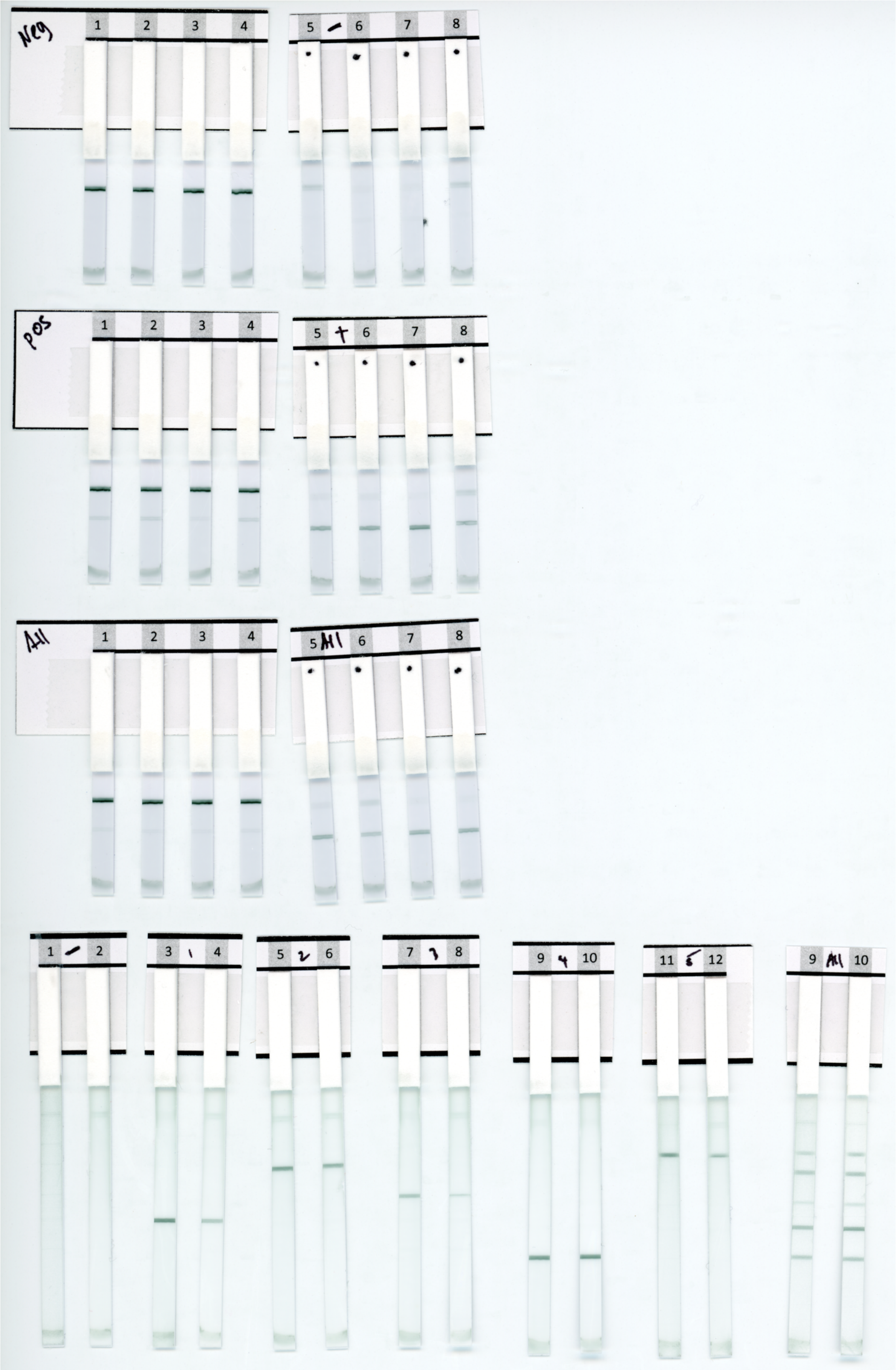

Unprocessed lateral flow strips for Fig. 5, replicate 1.

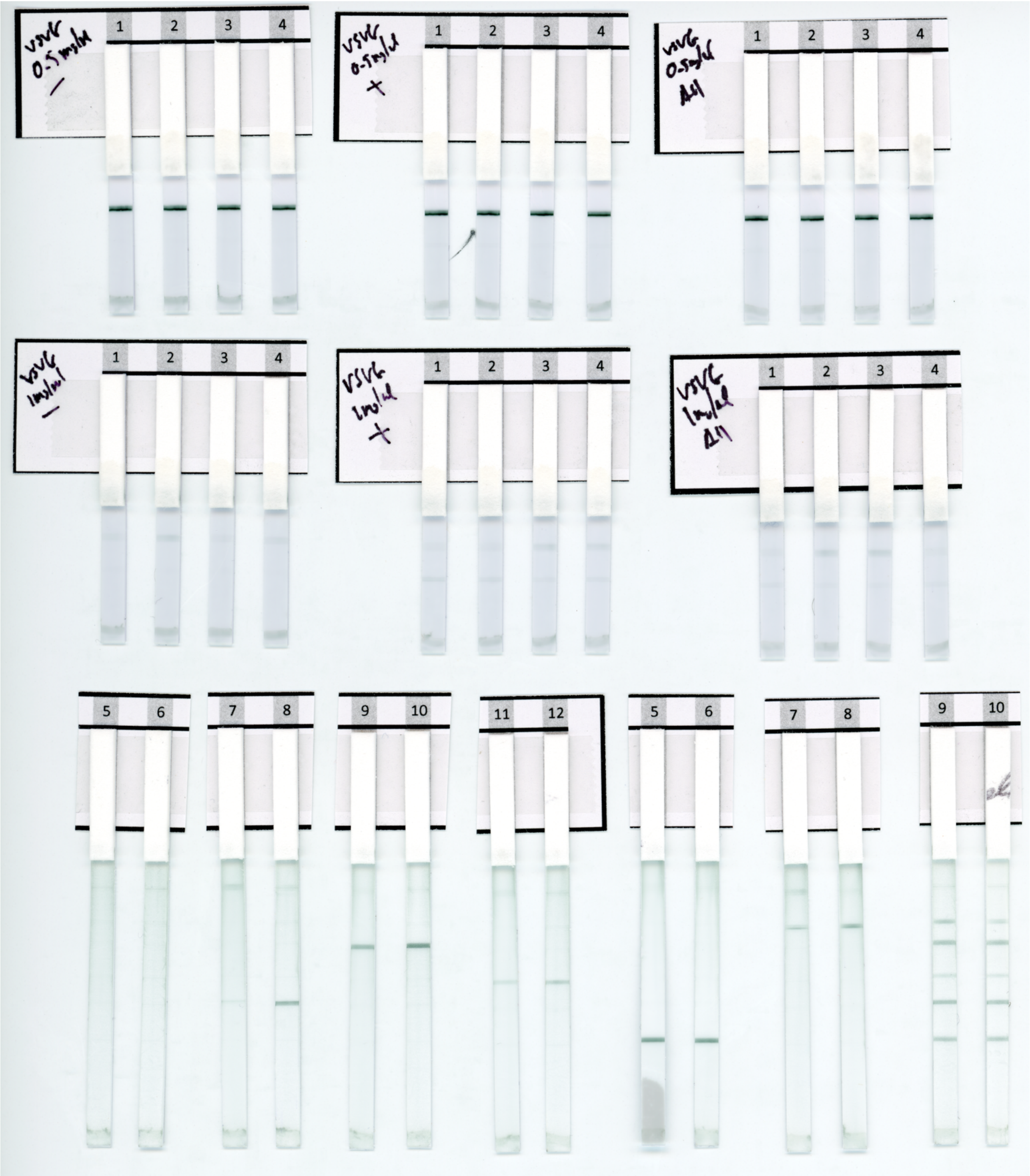

Unprocessed lateral flow strips for Fig. 5, replicate 2.

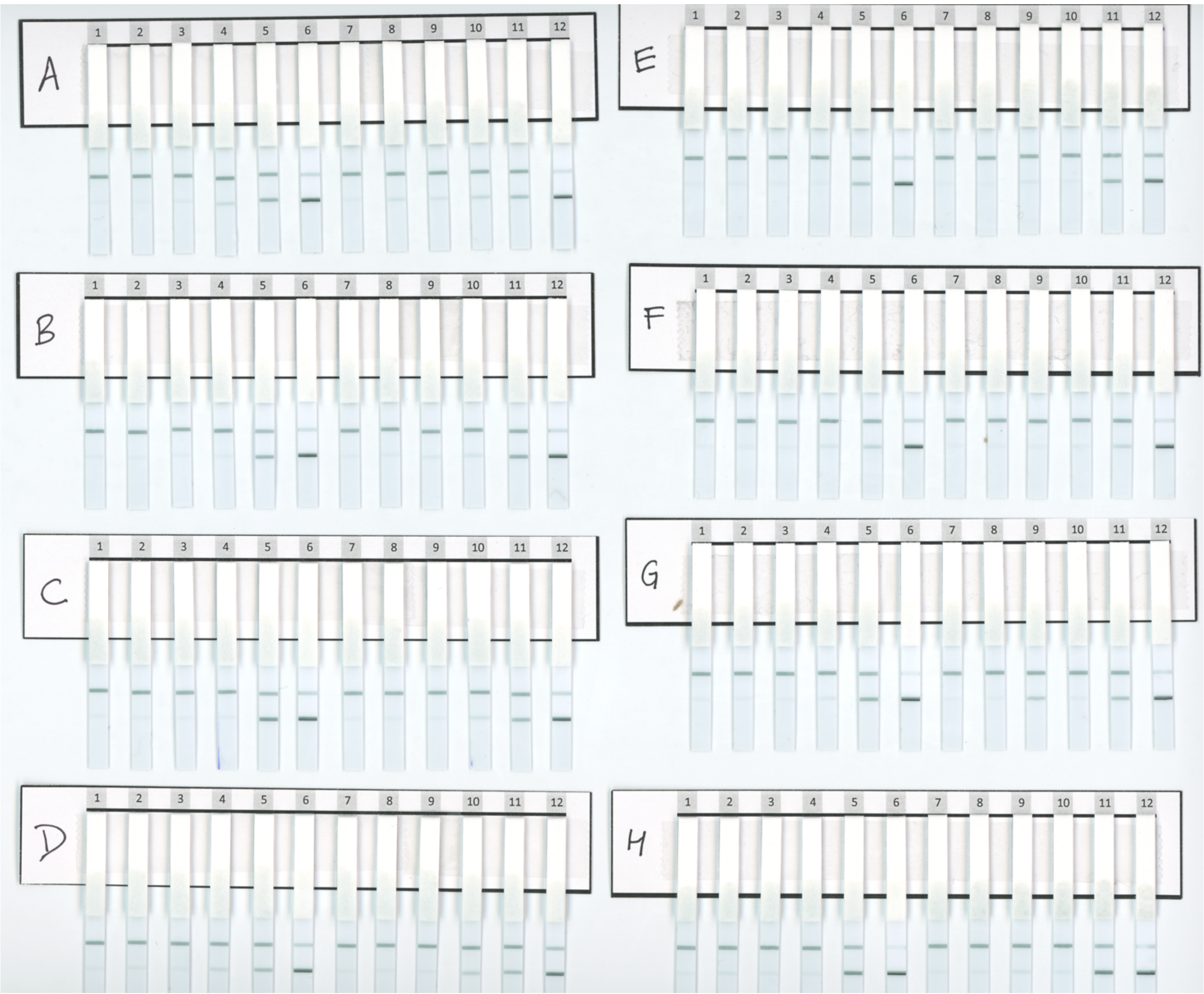

Unprocessed lateral flow strips for Fig. 6b.

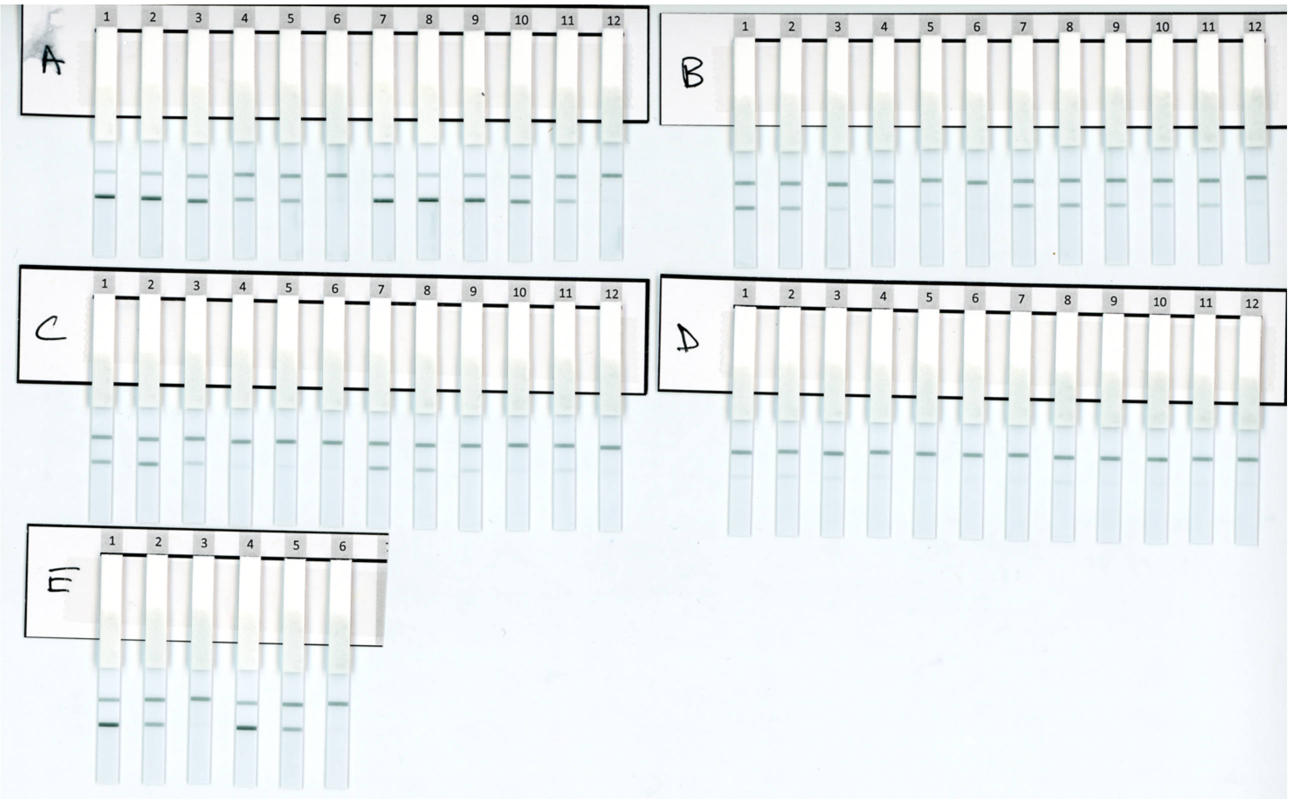

Unprocessed lateral flow strips for Fig. 6c.
