# Supplementary material for: Detection of viral RNAs at ambient temperature via reporter proteins produced through the target-splinted ligation of DNA probes
Source: Nat Biomed Eng. 2023 May 4;7(12):1571–82. doi: 10.1038/s41551-023-01028-y (PMC10727988; doi:10.1038/s41551-023-01028-y)
Supplement: Supplementary file 2 — Reporting Summary [file 41551_2023_1028_MOESM2_ESM.pdf]

nature portfolio

Corresponding author(s): Aric Joneja

Last updated by author(s): Mar 12, 2023

Reporting Summary

Nature Portfolio wishes to improve the reproducibility of the work that we publish. This form provides structure for consistency and transparency in reporting. For further information on Nature Portfolio policies, see our Editorial Policies and the Editorial Policy Checklist.

Statistics

For all statistical analyses, confirm that the following items are present in the figure legend, table legend, main text, or Methods section.

n/a	Confirmed	
☐	☒	The exact sample size (n) for each experimental group/condition, given as a discrete number and unit of measurement
☐	☒	A statement on whether measurements were taken from distinct samples or whether the same sample was measured repeatedly
☐	☒	The statistical test(s) used AND whether they are one- or two-sided<br>*Only common tests should be described solely by name; describe more complex techniques in the Methods section.*
☒	☐	A description of all covariates tested
☐	☒	A description of any assumptions or corrections, such as tests of normality and adjustment for multiple comparisons
☐	☒	A full description of the statistical parameters including central tendency (e.g. means) or other basic estimates (e.g. regression coefficient) AND variation (e.g. standard deviation) or associated estimates of uncertainty (e.g. confidence intervals)
☐	☒	For null hypothesis testing, the test statistic (e.g. F, t, r) with confidence intervals, effect sizes, degrees of freedom and P value noted<br>*Give P values as exact values whenever suitable.*
☒	☐	For Bayesian analysis, information on the choice of priors and Markov chain Monte Carlo settings
☒	☐	For hierarchical and complex designs, identification of the appropriate level for tests and full reporting of outcomes
☒	☐	Estimates of effect sizes (e.g. Cohen's d, Pearson's r), indicating how they were calculated

Our web collection on statistics for biologists contains articles on many of the points above.

Software and code

Policy information about availability of computer code

Data collection	Absorbance and luminescence values were collected via Biotek Neo2 with Gen 5 software (ver. 3.08). Lateral-flow test strips were imaged on an Epson V850 Pro scanner with EPSON Scan software (ver. 3.9.3.4).
Data analysis	ImageJ Version 1.53, Microsoft Excel 365 Version 2206, GraphPad Prism 9.

For manuscripts utilizing custom algorithms or software that are central to the research but not yet described in published literature, software must be made available to editors and reviewers. We strongly encourage code deposition in a community repository (e.g. GitHub). See the Nature Portfolio guidelines for submitting code & software for further information.

Data

Policy information about availability of data

All manuscripts must include a data availability statement. This statement should provide the following information, where applicable:

- Accession codes, unique identifiers, or web links for publicly available datasets
- A description of any restrictions on data availability
- For clinical datasets or third party data, please ensure that the statement adheres to our policy

All data needed to evaluate the conclusions can be found in the paper and its supplementary information. Source data are provided with this paper.

Human research participants

Policy information about studies involving human research participants and Sex and Gender in Research.

Reporting on sex and gender	The study did not involve human research participants.
Population characteristics	—
Recruitment	—
Ethics oversight	—

Note that full information on the approval of the study protocol must also be provided in the manuscript.

Field-specific reporting

Please select the one below that is the best fit for your research. If you are not sure, read the appropriate sections before making your selection.

☒ Life sciences ☐ Behavioural & social sciences ☐ Ecological, evolutionary & environmental sciences

For a reference copy of the document with all sections, see nature.com/documents/nr-reporting-summary-flat.pdf

Life sciences study design

All studies must disclose on these points even when the disclosure is negative.

Sample size	No sample-size calculation was performed; replicates or triplicates were deemed to be sufficient on the basis of previous observations of variability in the systems.
Data exclusions	No data were excluded.
Replication	The data provided in the paper are representative of the results recorded over many days and experiments, with similar (yet not identical) processes and outcomes.
Randomization	Only one variable (typically, target concentrations) was introduced into each experiment.
Blinding	The results of the analyses were objective and quantifiable, and not subject to investigator bias.

Reporting for specific materials, systems and methods

We require information from authors about some types of materials, experimental systems and methods used in many studies. Here, indicate whether each material, system or method listed is relevant to your study. If you are not sure if a list item applies to your research, read the appropriate section before selecting a response.

Materials & experimental systems

n/a	Involved in the study	
☐	☒	Antibodies
☒	☐	Eukaryotic cell lines
☒	☐	Palaeontology and archaeology
☒	☐	Animals and other organisms
☒	☐	Clinical data
☒	☐	Dual use research of concern

Methods

n/a	Involved in the study	
☒	☐	ChIP-seq
☒	☐	Flow cytometry
☒	☐	MRI-based neuroimaging

Antibodies

Antibodies used	anti-Strep-tag IIantibody (clone 5A9F9, A01732, Genscript, lot H2012001)<br>anti-FLAG test line (clone M2.1, cAb6404-1.1, Absolute Antibody, lots T2112B21, T2112B18)<br>Anti-HA (clone 5E11D8, cat. A01244-100, Genscript, lot 19L002034)<br>anti-Ty1 (clone BB2, cat. MA5-23513, ThermoFisher, lots WK3436911, WK3234861, and WK3440671)<br>anti-OLLAS (clone L2, cat. NBP1-0673, NovusBio, lots F-15 and F-16)<br>anti-V5 (clone SV5-Pk1, cat. R960-25, Invitrogen, lot 2330339)

anti-S-tag (clone GT727, cat. SAB2702227, Sigma Aldrich, lot 41295)
anti-VSV-G (clone P5D4, cat. ab50549, Abcam, lot GR34014830-5)
anti-Myc (clone 9E10, cat. M4439, Sigma Aldrich, lot 0000129827)
anti-mouse (polyclonal, cat. A28174, Thermo Fisher, lots 2324159 and 2352345)

Validation

Antibodies were validated by the manufacturers by ELISA. We also validated each antibody by ELISA and in a lateral-flow immunoassay format in which antibodies were striped onto a test membrane or conjugated to 40-nm gold nanoparticles. An antibody was considered valid if a test line appeared when subject to a synthesized peptide; no test line appeared if no peptide or off-target peptides were present.
